# High or low calcium intake increases cardiovascular disease risks in older patients with type 2 diabetes

**DOI:** 10.1186/s12933-014-0120-0

**Published:** 2014-08-01

**Authors:** Jui-Hua Huang, Leih-Ching Tsai, Yu-Chen Chang, Fu-Chou Cheng

**Affiliations:** Department of Community Health, Chia-Yi Christian Hospital, Chia-Yi, Taiwan; Division of Endocrine and Metabolism, Department of Internal Medicine, Erlin-Branch, Changhua Christian Hospital, Changhua, Taiwan; Department of Geriatric Medicine, Chia-Yi Christian Hospital, Chia-Yi, Taiwan; Stem Cell Center, Department of Medical Research, Taichung Veterans General Hospital, Taichung, Taiwan

**Keywords:** Calcium, Magnesium, Inflammation, Cardiovascular disease risk, Older patients with diabetes

## Abstract

**Background:**

We investigated the effects of dietary calcium (Ca) and magnesium (Mg) intakes on cardiovascular disease risks in older patients with diabetes.

**Methods:**

In this cross-sectional study, 197 patients with type 2 diabetes aged 65 years and above were recruited. The 24-h dietary recalls and 1-week self-reported typical dietary intake patterns were collected. The Ca and Mg intakes of <67% of the recommended dietary allowance (RDA), 67%–100% of RDA, and >100% of RDA were defined as low, moderate, and high Ca and Mg intakes, respectively. Anthropometric measurements were determined and biochemical analysis of blood and urine was performed.

**Results:**

Our data indicated that 60.9% and 87.3% of our patients were Ca and Mg intakes below RDA, respectively. Patients whose Ca intake was high or low (81.2%) had significantly higher C-reactive protein (CRP) than those whose Ca intake was moderate (*p =* 0.043). Furthermore, patients whose Mg intake was low (87.3%) had significantly higher CRP than that of those who took adequate Mg (*p* = 0.025). The dietary Ca:Mg intake ratios were highly correlated with CRP, platelet counts, and red blood cell distribution (*p* < 0.05). A dietary Ca:Mg intake ratio of 2.0–2.5 was significantly correlated to lower CRP levels (*p =* 0.013).

**Conclusions:**

High or low calcium intake increases cardiovascular disease risks. We suggest that “moderate” intake of 402–600 mg Ca/day (approximately 67%–100% of Taiwan RDA for Ca) and adequate Mg intake (or meeting RDA for Mg) with Ca:Mg intake ratio of 2.0–2.5 are important for reducing cardiovascular disease risks in older patients with diabetes.

## Background

Diabetes is frequently associated with cardiovascular disease (CVD) and cardiovascular complications [1]. In general, patients with diabetes have high risk for CVD, a major death factor, particularly in older patients [2,3]. Inflammation also plays a key role in the pathogenesis and progression of CVD in patients with diabetes [4–6]. Diet, lifestyle, and medical interventions may promote or retard inflammatory responses in diabetic management [7–9]. Low magnesium (Mg) and calcium (Ca) intakes raise inflammatory and CVD risks [10–12]. However, the effects of high Ca or Mg intakes on cardiovascular risks in patients with diabetes remain unclear.

Low Mg intakes are associated with increased inflammation and risk of type 2 diabetes and CVD [12–14]. A nationally representative cross-sectional survey showed that adults in the USA consuming Mg below the recommended dietary allowances (RDAs) were more likely to have elevated C-reactive protein (CRP), which may contribute to CVD risks [12]. In rodents, the intake of an Mg-deficient high-fat diet led to alterations in the insulin-signaling pathway and increased insulin resistance [15]. In patients with diabetes, low serum Mg levels were found to be associated with macrovascular complication [16], and hypomagnesemia was arryhtmogenic [17]. In addition, low serum Mg levels increased chronic inflammatory stress that could be alleviated by increasing Mg intakes in middle-aged people with poor quality of sleep [18]. Dietary Ca intakes were also reported to be associated with the modulation of inflammatory stress and pathogenesis of CVD [11,19,20]. However, whether high Ca intake decreases or increases the risk of CVD remains controversial [11,20]. Zemel et al. [19] found that dietary Ca suppresses oxidative and inflammatory stress. A cross-sectional study showed that increased Ca intakes decreased the risk of several CVD risk factors [21]. In contrast, in another study, increasing dietary Ca intake or use of Ca supplementation was noted to raise myocardial infarction risks [22]. Although Mg and Ca intakes are closely related to inflammation and CVD risks, they also interact and each antagonizes the other’s absorption in the intestinal tract [23]. Mg is a natural physiologic Ca antagonist. Therefore, varied Mg and Ca dietary intakes can alter the absorption of Mg or Ca alone, and inappropriate Mg and Ca dietary intakes can cause additional CVD risks.

The intestinal absorption of Ca or Mg may depend on the amounts of Ca and Mg present in the diet [23]. Therefore, owing to the nature of these intestinal interactions, the dietary Ca:Mg intake ratio and the absolute amount of Ca or Mg in the daily diet may be equally important in reducing CVD risks. Recently, Dai et al. found that the impact of Ca:Mg intake ratio on the risk of CVD had significantly higher modifying effects than that of Ca or Mg intake alone in Chinese populations [24]. In addition, the United States Department of Agriculture (USDA) food surveys from 1977 through 2008 showed a rise in the Ca:Mg intake ratio, which coincided with the increased prevalence and incidence of type 2 diabetes [25]. Therefore, the effects of dietary Ca and Mg intakes on CVD risks may vary depending on the dietary Ca:Mg intake ratio. Thus, the roles of dietary Ca and Mg intakes and their ratio in the prevention of CVD warrant further investigation.

Older patients with diabetes are at high risk for CVD [2,3], and Mg deficiency is common in this population [26,27]. Mg deficiency may be exacerbated by low Mg with high or low Ca intakes [13,28], which may trigger inflammatory stress, contributing to the development of CVD [13]. However, the effects of dietary Ca and Mg intakes on CVD risk have not been intensively explored in older patients with diabetes. To date, numerous studies have demonstrated correlations between Ca or Mg intakes alone with CVD risk [10,29,30], but relatively few studies have examined the potentially combined effects of dietary intakes of Ca and Mg on CVD risk [24]. The present study was conducted to explore CVD risks in older patients with type 2 diabetes with respect to their dietary Mg and Ca intakes and Ca:Mg intake ratios. Dietary intakes and lifestyle data were obtained and assessed using questionnaires. In addition, anthropometric measurements were determined and biochemical analysis of blood and urine samples was performed.

## Methods

### Study design

The investigation employed a cross-sectional research design targeting patients with type 2 diabetes aged 65 years and above. Diabetes was diagnosed in the Endocrine and Metabolism Clinic according to the guidelines of the American Diabetes Association (ADA-1997) [31]. The inclusion criteria for patients were as follows: *1*) type 2 diabetes for >6 months; *2*) no change in any medications for the past 3 months; *3*) stable lifestyle for the past 3 months; and *4*) absence of heart failure, cirrhosis, current malignancy, chronic renal failure, or clinically relevant infection (CRP levels > 10 mg/L). Patients with signs of serious deterioration in comprehension and memory were excluded. A total of 197 patients were included in the study. This investigation was performed in compliance with the Helsinki Declaration, and approved by the Changhua Christian Hospital Institutional Review Board (CCHIRB: 090419).

### Assessment of dietary intakes, lifestyle, and body mass index

Dietary intakes were assessed using 24-h recall and 7-day typical dietary intake by interview and dietary records [32,33]. During the interview, quantitative tools including standard measuring spoons and cups, food models, food pictures and photos, as well as traditional household bowls, cups, and spoons were used to help the elderly subjects properly estimate their dietary intake [34]. Intakes of Ca, Mg, and other nutrients were analyzed using Taiwan’s Nutrition Database and the EKitchen nutritional analysis software (Nutritional Chamberlain Line, Professional Edition, version 2001/2003, EKitchen Inc, Taichung, Taiwan) [35]. In addition, data on potential confounders, such as lifestyle factors, including physical activity, smoking, and alcohol consumption, were collected using a self-reported questionnaire. Anthropometric measurements included height and weight. Body mass index (BMI) was calculated as weight (kg)/height (m^2^).

Moderate nutrient intakes were defined as consumption of 67% of RDA for that nutrient or 67% of its adequate intake value (AI) [36]. In the present study, assessment of Ca intakes was based on a previous RDA for Ca for Taiwanese aged 65 years and above (600 mg/day), because the Ca intake level for majority of older patients with diabetes was below the current AI for Ca (1000 mg/day). The Ca intakes were categorized as follows: 1) low: Ca intakes <67% of RDA for Ca; 2) moderate: Ca intakes approximately 67%–100% of RDA for Ca; and 3) high: Ca intakes more than RDA for Ca [36]. In addition, assessment of Mg intakes was based on RDA for Mg for Taiwanese aged 65 years and above (350–360 mg/day for older men and 300–310 mg/day for older women). The Mg intakes were categorized as follows: 1) low: Mg intakes <67% of RDA for Mg, 2) moderate: Mg intakes approximately 67%–100% of RDA for Mg, and 3) high: Mg intakes more than RDA for Mg [36].

### Markers of inflammation and CVD risks

Inflammatory markers were measured by the hospital medical laboratory (certified ISO15189) to assess CVD risk and included high-sensitivity CRP [37], leukocyte counts [38], platelet counts [39], and red blood cell distribution width (RDW) [40]. The high-sensitivity CRP (CV <3.0%) was measured by particle-enhanced turbidimetric immunoassay (Dimension, Siemens, Newark, USA). The CRP levels of <1, 1–3, and >3 mg/L represented low, moderate, and high CVD risk, respectively [37]. Patients with clinically relevant infection (CRP levels >10 mg/L) were excluded. In addition, blood leukocytes (CV <3.0%) and platelets (CV <3.0%) were measured by the direct current detection method (XT1800i, Sysmex, Kobe-shi, Hyogo, Japan) and RDW was calculated. The subjects were stratified into tertiles (low, medium, and high) based on leukocyte counts, platelets, and RDW.

### Renal functional measurements

The serum creatinine levels (CV <2%) were determined by the alkaline picrate-kinetic method. The estimated glomerular filtration rate (GFR) was calculated as eGFR (mL/min/1.73 m^2^) (Simplified Modification of Diet in Renal Disease (MDRD)) = 186 × serum creatinine^−1.154^ × Age^−0.203^ for men and 186 × serum creatinine^−1.154^ × Age^−0.203^ × 0.742 for women, according to the formula recommended by the Taiwan Society of Nephrology [41].

### Statistical analysis

The categorical variables were analyzed by the chi-square test, and the data are presented in number (n) and percent (%). For continuous dependent variables, comparisons of the means were analyzed by 2-tailed t-test (2 groups) or one-way ANOVA followed by Scheffe’s multiple comparisons test, and the data are presented as mean ± SD. In addition, the correlations of dietary Ca and/or Mg intakes and markers of inflammation with CVD risks were examined by multivariate analyses, followed by Bonferroni’s multiple comparisons test, and the data are presented as adjusted mean ± standard error (SE). All the statistical procedures were performed using SPSS 17.0 statistical software (SPSS Inc., Chicago, IL, USA), and a *p* value <0.05 was considered statistically significant.

## Results

The characteristics of the 197 older patients with type 2 diabetes are summarized in Table 1. Among all the patients, 60.9% of the patients’ Ca intake was less than the previous RDA (600 mg/day) and 87.3% patients’ Ca intakes were below the current AI (1000 mg/day). In addition, 87.3% of the patients’ Mg intake was below RDA, and the total energy, carbohydrate, fat, and Mg intakes were different between men and women (*p* ≦ 0.05). The analyses were adjusted for potential confounders, which included lifestyle factors such as physical activity, smoking, and alcohol consumption (data not shown).

**Table 1:** **Characteristics of older adults with type 2 diabetes by gender**

**Variables**	**Total (n = 197)**	**Male (n = 90)**	**Female (n = 107)**	***P*** ^**4**^
Age (y)	72.1 ± 5.3	72.2 ± 5.4	72.1 ± 5.3	0.935
Diabetes medication				
Oral hypoglycemic drug	138 (70.1)	65 (72.2)	73 (68.2)	0.542
Insulin and oral hypoglycemic drug	69 (29.9)	25 (27.6)	34 (31.8)	
Glycated hemoglobin (%)	7.3 ± 1.3	7.4 ± 1.3	7.3 ± 1.3	0.534
Estimated GFR^1^ (ml/min)	71.2 ± 19.1	70.3 ± 19.1	71.9 ± 19.1	0.541
Body mass index (kg/m^2^)	25.3 ± 3.7	24.9 ± 3.4	25.6 ± 4.0	0.176
High-sensitivity CRP^1^ (mg/L)				
<1 low cardiovascular risk	57 (28.9)	27 (30.0)	30 (28.0)	0.169
1–3 moderate cardiovascular risk	105 (53.3)	52 (57.8)	53 (49.5)	
>3 high cardiovascular risk	35 (17.8)	11 (12.2)	24 (22.4)	
Dietary intake				
Total energy intake (kcal/day)	1583.7 ± 414.3	1765.3 ± 434.5	1424.8 ± 322.1	<0.001
Carbohydrate intake (% of energy)	60.9 ± 8.5	59.5 ± 8.5	62.1 ± 8.3	0.027
Protein intake (% of energy)	12.4 ± 2.6	12.5 ± 2.4	12.3 ± 2.8	0.464
Fat intake (% of energy)	26.7 ± 7.3	28.2 ± 7.5	25.3 ± 6.9	0.004
Calcium (mg/day)	556.9 ± 385.3	557.9 ± 319.1	556.0 ± 436.5	0.972
<AI	172 (87.3)	82 (91.1)	90 (84.1)	0.142
<a previous RDA^1,2^	120 (60.9)	53 (58.9)	67 (62.6)	0.593
Magnesium (mg/day)	218.4 ± 102.4	252.5 ± 112.8	188.6 ± 81.8	<0.001
<RDA^3^	172 (87.3)	72 (80.0)	100 (93.5)	0.005

^1^GFR: glomerular filtration rate, CRP: C-reactive protein, AI: adequate intake, RDA: recommended dietary allowance, EAR: estimated average requirement.

^2^A previous Taiwan RDA for Ca for healthy individuals above 65 years of age is 600 mg/day. Current Taiwan AI of Ca for healthy individuals above 65 years of age is 1000 mg/day.

^3^Taiwan RDA for Mg for health individuals above 65 years of age is 350–360 mg/day for men and 300–310 mg/day for women.

^4^A *p* value <0.05 was considered statistically significant. Continuous data are presented as mean ± SD. Categorical data are presented as number (n) and percent (%).

As shown in Table 2, the dietary Ca:Mg intake ratio was significantly associated with the levels of CRP, platelets, and RDW (*p* < 0.05). The subgroup with a Ca:Mg intake ratio of 2.0–2.5 had significantly lower CRP and RDW than the one with a Ca:Mg intake ratio of >3.6. Moreover, the subgroup with a Ca:Mg intake ratio of 2.0–2.5 had a lower proportion of patients with ≥2 high inflammatory markers, when compared with the other subgroups. However, the dietary Ca:Mg intake ratio was not significantly associated with leukocyte count. In addition, the subgroup with a Ca:Mg intake ratio of 2.0–2.5 presented a significantly higher Ca intake than those with Ca:Mg intake ratios of ≤1.3 and 1.4–1.9. In contrast, the subgroup with a Ca:Mg intake ratio of 2.0–2.5 exhibited a significantly lower Ca intakes than the one with a Ca:Mg intake ratio of >3.6. Moreover, the dietary Ca:Mg intake ratio showed a marginal correlation with Mg intakes (*p =* 0.099).

**Table 2:** **Relationships of dietary Ca:Mg intake ratio and markers of inflammation and CVD risk in older patients with diabetes**

	**Quintiles of dietary Ca:Mg ratio**					
**Variables**	**≤1.3 (n = 40)**	**1.4–1.9 (n = 39)**	**2.0–2.5 (n = 42)**	**2.6–3.6 (n = 38)**	**>3.6 (n = 38)**	***p*** ^**4**^
Dietary Ca and Mg intake^1^						
Calcium intake (mg/day)*	212.9 ± 179	312.8 ± 158.2	555.3 ± 219.0	700.6 ± 261.1	1060 ± 396.5	<0.001
Magnesium intake (mg/day)	223.9 ± 138.6	204.3 ± 96.9	251.6 ± 104.0	231.1 ± 90.7	193.7 ± 64.8	0.099
Markers of inflammation and cardiovascular risk^2^						
High-sensitivity CRP (mg/L)^†^	1.8 ± 0.3	1.9 ± 0.4	1.2 ± 0.4	1.8 ± 0.4	2.7 ± 0.4	0.013
White blood cell (10^9^ cells/L)	6.6 ± 0.3	6.2 ± 0.3	5.9 ± 0.3	6.8 ± 0.4	6.4 ± 0.4	0.200
Platelet (10^3^/μL)	227.5 ± 11.0	203.4 ± 11.2	205.7 ± 11.3	235.5 ± 11.7	224.3 ± 11.7	0.046
Red blood cell distribution width (%)^†^	13.5 ± 0.2	13.6 ± 0.2	13.1 ± 0.2	13.7 ± 0.2	13.8 ± 0.2	0.032
Number of high inflammatory markers^3^						
0	10(25.0)	17(43.6)	21(50.0)	12(31.6)	7(18.4)	0.002
1	14(35.0)	7(17.9)	18(42.9)	10(26.3)	16(42.1)	
≥2	16(40.0)	15(38.5)	3(7.1)	16(42.1)	15(39.5)	

^1^The comparisons of the means were analyzed by one-way ANOVA. *indicates significant difference in Ca intake between the subgroup with a ratio of 2.0–2.5 and those with ratios of ≤1.3, 1.4–1.9, and >3.6 by Scheffe’s multiple comparisons test.

^2^The analyses were adjusted for sex, age, glycated hemoglobin, BMI, physical activity levels, smoking, alcohol consumption, and total energy, carbohydrate, protein, and fat intake. The data are adjusted mean ± standard error (SE). ^†^indicates significant difference in CRP and red blood cell distribution width between the subgroup with a ratio of 2.0–2.5 and the one with a ratio of >3.6 by Bonferroni’s multiple comparisons test.

^3^Categorical variables were analyzed by chi-square test. The data are presented as number (n) and percent (%). The definition of high levels of the following four inflammatory markers: CRP >3 (mg/L), white blood cell >6.5 (10^3^/mm^3^), platelet >227.0 (10^3^/μL), and red blood cell distribution width >13.6 (%).

^4^A *p* value of <0.05 was considered statistically significant.

**Figure 1:**
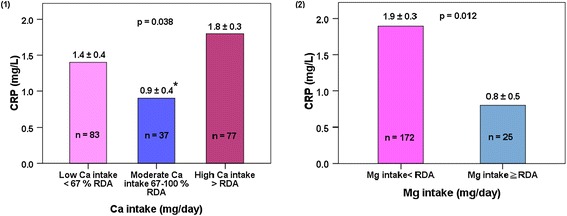
**Relationship between CRP and Ca or Mg intake alone. (1)** CRP and Ca intakes. **(2)** CRP and Mg intakes. The analyses were adjusted for sex, age, BMI, physical activity levels, smoking, alcohol consumption, total energy, carbohydrate, protein and fat intakes, and Mg intakes for different levels of Ca intakes or Ca intakes for different levels of Mg intakes. Data are adjusted mean ± standard error (SE). A *p* value of <0.05 was considered statistically significant. *indicates significant differences between moderate and high Ca intakes by Bonferroni’s multiple comparisons test.

The relationships between CRP and dietary Ca or Mg intake alone are shown in Figure 1. Dietary Ca intake was significantly correlated to the CRP level (*p =* 0.038). Moreover, patients consuming moderate amount of Ca (402–600 mg Ca/day) had lower CRP than those consuming high amount of Ca, i.e. >600 mg Ca/day (0.9 ± 0.4 vs. 1.8 ± 0.3, *p* = 0.033). In addition, patients consuming high or recommended amount of Mg (RDA for Mg for healthy men and women in Taiwan above 65 years of age is 350–360 and 300–310 mg/day, respectively) had a lower CRP level than those consuming low amount of Mg (0.8 ± 0.5 vs.1.9 ± 0.3, *p* = 0.012).

The correlations between different CRP levels (low, moderate, and high CVD risks) and different Ca and Mg intake levels (nine subgroups) were analyzed by chi-square test. The findings showed that CRP levels were correlated to dietary Ca and Mg intakes. Although some cells had an expected count of <5, the Chi-square test showed a statistically significant result (*p* = 0.001). The above-mentioned nine subgroups with different levels of Ca and Mg intakes were categorized as follows: 1) low Ca and low Mg intakes; 2) low Ca and moderate Mg intakes; 3) low Ca and high Mg intakes; 4) moderate Ca and low Mg intakes; 5) moderate Ca and moderate Mg intakes; 6) moderate Ca and high Mg intakes; 7) high Ca and low Mg intakes; 8) high Ca and moderate Mg intakes; and 9) high Ca and high Mg intakes. In addition, the correlations between different CRP levels (low, moderate, and high CVD risks) and dietary Ca:Mg intake ratio were examined by one-way ANOVA. The results showed that the dietary Ca:Mg intake ratios in the low, moderate, and high CVD risk groups were 2.1 ± 1.3, 2.8 ± 1.8, and 3.0 ± 1.8, respectively (*p* = 0.016).

**Figure 2:**
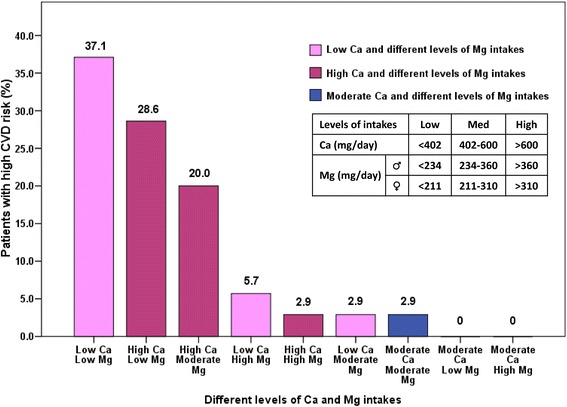
**Distribution of high CVD risk patients according to Ca and Mg intakes.** CRP >3 mg/L defined as high CVD risk. Data are presented as percent (%).

The distribution of patients with high CVD risk (CRP >3 mg/L) according to different levels of Ca and Mg intakes (nine subgroups) is presented in Figure 2. Among the patients with high CVD risk, 37.1% had low Ca and low Mg intakes, 28.6% had high Ca and low Mg intakes, 20.0% had high Ca and moderate Mg intakes, and 5.7% had low Ca and high Mg intakes. In addition, the high Ca and high Mg intakes subgroup, moderate Ca and moderate Mg intakes subgroup, and low Ca and moderate Mg intakes subgroup each comprised 2.9% of the patients with high CVD risk. No patients with high CVD risk consumed a diet with moderate amount of Ca and low or high amount of Mg. Furthermore, 97.1% of the high CVD risk patients were found to consume high or low amount of Ca and only 2.9% consumed moderate amount of Ca, even when these patients were stratified by Mg intakes. Among the high CVD risk patients who consumed low or high amount of Ca, the percentage of patients who consumed low amount of Mg was higher than that of patients who consumed moderate to high amount of Mg (65.7% vs. 31.5%).

## Discussion

The effects of high or low Ca and low Mg intakes on CVD risks in patients with diabetes have not been fully elucidated. This cross-sectional study investigated the effects of dietary Ca and Mg intakes on CVD risk in older patients with type 2 diabetes. Our data showed that (1) the Ca intake in 60.9% of the patients was lower than the previous RDA and the Mg intake in 87.3% of the patients was below RDA; (2) high or low Ca intake may cause high CVD risks; (3) low Mg intakes were correlated to high CVD risk; and (4) dietary Ca:Mg intake ratio of 2.0–2.5 was correlated to low CVD risks.

### Ca and Mg intakes in the older population

Ca and Mg are essential elements in human physiology and are especially important in the biological functions of the cardiovascular system [30,42]. However, in many countries, it has been reported that the dietary Ca and Mg intakes in the general population remain below RDA, particularly among the elderly [11,43,44]. In general, RDA for Ca for healthy individuals above 65 years of age is 650–1300 mg/day [11,43,45]. In our data, the daily consumption of Ca in older men and women with diabetes was only 558 and 556 mg, respectively. Furthermore, 60.9% of the older patients with diabetes did not meet the previous RDA for Ca (600 mg/day), and 87.3% of the patients did not meet the current AI for Ca (1000 mg/day). The data from the National Health and Nutrition Examination Survey (NHANES) 2005–2008 indicate that most of the Americans over 50 years of age (92–93%) did not meet the AI for Ca (1200 mg/day) [43]. Furthermore, the data from the Nutrition and Health Surveys in Taiwan (NAHSIT) 2005–2008 show that the average Ca intakes in men and women over 50 years of age were 673 and 592 mg, respectively [46], suggesting that 82%–94% of Taiwanese over 65 years of age, regardless of gender, did not meet the previous RDA for Ca (600 mg/day) [46]. Moreover, it has been reported that dietary Mg intakes below RDA are common in populations throughout the world [43,44]. The general RDA for dietary Mg for healthy individuals above 65 years of age is 310–420 mg/day for men and 270–320 mg/day for women [43–45]. In the present study, the average Mg intakes in older men and women with diabetes were 253 and 189 mg, respectively. Furthermore, our data showed that the Mg intake in 80% of older men and 94% of older women with diabetes was lower than RDA. The data from the NAHSIT 2005–2008 showed that the average Mg intakes in men and women over 65 years were 279 and 227 mg, respectively [46]. These findings indicate that 87% of elderly men and 93% of elderly women did not meet RDA for Mg [46]. Furthermore, the data from the NHANES 2005–2008 showed that the majority of the Americans over 50 years of age (55%–70%) did not meet the estimated average requirement (EAR) for Mg [43]. The data obtained in the present study suggest that, similar to most of the elderly people, the intakes of Ca and Mg in the majority of older patients with diabetes are low. Thus, recommendation for improvements in the dietary Ca and Mg intakes to achieve adequate intakes for the purpose of reducing CVD risks is important for older patients with diabetes.

### Inappropriate Ca intake and CVD risk

Inappropriate Ca intake may be linked to triggering of an inflammatory response, which has been implicated in the pathogenesis of CVD [11]. Low Ca intake affects the development and outcome of CVD [47]. However, the effect of increased Ca intakes by consuming dietary Ca or Ca supplements on CVD risks remains controversial [10,11,22]. The data obtained in the present study showed that older patients with diabetes consuming diets with low level of Ca had higher CRP level, which may contribute to high CVD risks. Moreover, one of the major findings of the present study is that diets with high level of Ca may also cause high CVD risks unless consumed with an adequate amount of Mg (Mg intakes ≥ RDA for Mg; Taiwan RDA for Mg for healthy individuals above 65 years of age is 350–360 mg/day for men and 300–310 mg/day for women). Conversely, intake of diets with moderate amount of Ca reduces CVD risks in older patients with diabetes. Zemel et al. indicated that dietary Ca suppresses oxidative and inflammatory stress [19]. Another study demonstrated that increased Ca intakes decreased the risk of CVD [21]. In contrast, a prospective study showed that an increase in the dietary Ca intakes or Ca supplements increased myocardial infarction risk [22]. Therefore, Ca may be a double-edged sword. A deficiency of Ca may evoke increased secretion of parathyroid hormone, which increases bone resorption, thereby removing Ca from the bones, and excess of Ca is associated with many inflammatory and degenerative diseases [47]. Thus, low Ca intakes may increase CVD risks and bone loss, whereas excess Ca intakes may lead to Ca deposition in the arteries or vascular calcification, and could therefore increase the risks of CVD [11]. The findings of the present study and those from several previous studies suggest that high or low Ca intakes may increase the risks of CVD. Therefore, it is important to recommend a diet with moderate amount of Ca to reduce CVD risks in older patients with diabetes. It might be beneficial to suggest a range corresponding to “moderate” Ca intakes of 402–600 mg/day (approximately 67%–100% of Taiwan RDA for Ca).

### Inadequate Mg intake and CVD risk

Low Mg intakes are also closely correlated to increased inflammation and CVD risks [12,13]. In the present study, patients whose Mg intake was below RDA had elevated CRP levels, which may be related to a high risk of CVD. A nationally representative cross-sectional survey showed that the Mg intakes in a total of 68% of the American adults were below RDA, which may result in increased CRP level and contribute to CVD risks [12]. In middle-aged people with poor quality of sleep, a low serum Mg level was reported to be correlated to increased chronic inflammatory stress that could be alleviated by increasing the Mg intake [18]. In the Chinese population with diabetes, patients with macrovascular complications had lower serum Mg level than those with no macrovascular complications [16], and hypomagnesemia was arryhtmogenic [17]. In rodents, the intake of Mg-deficient high-fat diet led to alterations in the insulin-signaling pathway and increased insulin resistance [15]. Moreover, diabetic rats showed extensive cardiac remodeling and decreased myofibrillar Ca sensitivity, consistent with the observed increases in the phosphorylation of troponin I [48]. Inadequate Mg intakes may cause a decrease in the extracellular Mg, leading to the influx of Ca into the cells, which could trigger the release of proinflammatory cytokines and acute phase proteins from leukocytes, macrophages, and adipocytes [28,49]. Proinflammatory cytokines are released into the bloodstream and promote the release of CRP from the liver, which could result in an inflammatory response, platelet aggregation, and endothelial dysfunction, and may ultimately contribute to the development of CVD and metabolic disorder [28,49]. The data obtained in the present study showed that low Mg intakes are associated with high CVD risks. Thus, improvements in the dietary Mg intakes should be recommended for older patients with diabetes to achieve RDA for Mg and thereby reduce CVD risks.

### Dietary Ca:Mg intake ratio and CVD risk

Systemic inflammatory activity plays a key role in the pathogenesis and progression of CVD and type 2 diabetes [4]. Inflammatory biomarkers may therefore be a valuable tool in the evaluation of CVD risk. Among the inflammatory markers, CRP is considered to be the most well-validated and standardized marker for the evaluation of CVD risks [37,50]. In addition, increased leukocyte count, platelets, and RDW are also correlated to inflammation and cardiovascular complications in patients with type 2 diabetes [38–40]. In the present study, we analyzed these inflammatory markers to determine the effects of dietary Ca and Mg intakes on CVD risks in older patients with type 2 diabetes. Our findings showed that a diet with a Ca:Mg intake ratio of <2.0 or >2.5 may increase the risks of CVD in older patients with diabetes. In contrast, patients maintaining a dietary Ca:Mg intake ratio of 2.0–2.5 had lower levels of CRP, leukocytes, platelets, and RDW. Moreover, in the group of patients with a Ca:Mg intake ratio of 2.0–2.5, there was a lower proportion of patients with ≥2 high inflammatory markers, when compared with the other groups. These findings are in line with the recent Chinese population based cohort study [24]. Dai et al. found that the Ca:Mg intake ratio had significant modifying effects on CVD risks, when compared with the intakes of Mg or Ca alone [24]. Among the participants with Ca:Mg intake ratios >1.7, the intakes of Ca and Mg were associated with reduced risks of total mortality and mortality due to coronary heart diseases. Conversely, among the participants with a Ca:Mg ratio ≤ 1.7, the intake of Mg was associated with increased risks of total mortality and mortality due to CVD [24]. The data from the USDA food surveys from 1977 through 2008 revealed that the dietary Ca intakes have increased significantly than the dietary Mg intakes. Furthermore, the Ca:Mg intake ratios were found to increase from <2–3 in 1995 to ≥3.0 after 2000, coinciding with a rise in the age-adjusted type 2 diabetes incidence from 3.3% to >4.5% and age-adjusted prevalence rate increase from 4.7% to >6.2% in the American population [25]. It has been suggested that the Ca:Mg intake ratio should not be >2.0 from both foods and supplements. This suggestion is consistent with one of our major findings that a dietary Ca:Mg intake ratio of 2.0–2.5 is optimal for reducing CVD risks in older patients with diabetes. The findings of the present study and those of some previous studies indicate that a Ca:Mg intake ratio between 1.7 and 2.5 may be required to reduce CVD risk. In general, inadequate Ca and/or Mg intakes are correlated to inflammation and CVD risk [11,28,49]. Our data suggest that dietary Ca:Mg intake ratio is related to the markers of inflammation and cardiovascular complications in older patients with diabetes. An optimal dietary Ca:Mg intake ratio for reducing CVD risks in older diabetes patient may be 2.0–2.5.

### Ca and Mg intakes and CVD risk

Ca interacts and naturally antagonizes Mg in the absorption from the intestinal tract into the bloodstream [23,51]. Therefore, different dietary Ca:Mg intake ratios alter the absorption of Mg or Ca alone, and diets with low amount of Mg and high or low amount of Ca may cause high CVD risk. Indeed, our data indicated that low Mg and high or low Ca intakes were more prevalent in our high CVD risk patients than moderate to high Mg and high or low Ca intakes (65.7% vs. 31.5%). Interestingly, our data also showed that the majority of these high CVD risk patients (97.1%) consumed high or low amount of Ca. In contrast, the percentage of high CVD risk patients whose Ca intake was moderate was only 2.9%. Furthermore, among the 25 patients whose Ca intakes were >1000 mg/day, only 4 had low CVD risks (CRP <1 mg/L). Among these 4 patients, 3 consumed high amount of Mg and maintained a dietary Ca:Mg intake ratio of 2.0, 2.7, and 3.3, respectively. Among the other 21 patients whose Ca intakes were >1000 mg/day and CRP >1 mg/L, 5 and 16 patients had dietary Ca:Mg intake ratios of 2.9–3.6 and >4.7. Moreover, our findings showed that dietary Ca:Mg intake ratios in the low, moderate, and high CVD risk groups were 2.1 ± 1.3, 2.8 ± 1.8, and 3.0 ± 1.8, respectively (*p* = 0.016), indicating that inappropriate Ca and/or Mg intakes and Ca:Mg intake ratios may increase inflammation and CVD risks. Older persons with diabetes are at high risk for CVD [2,3] and inadequate Mg intakes are common in older persons [27]. High or low Ca intake may intensify the response to subclinical Mg deficiency, leading to increased CVD risk [13,28]. The results obtained in the present study suggest that consumption of moderate amount of Ca and adequate amount of Mg as well as maintenance of a Ca:Mg intake ratio of 2.0–2.5 are important for reducing CVD risks in older patients with diabetes. In addition, if patients could achieve an adequate dietary Ca intake of approximately ≥1000–1200 mg/day by taking Ca supplements, then, to maintain a Ca:Mg intake ratio of 2.0–2.5 for reducing CVD risks, they may either have to decrease the Ca intake or increase the Mg intake.

### Policy implications for medical care

Our findings raise issues that may have policy implications for medical care in older patients with diabetes. It has been established that low Ca and/or low Mg intakes could increase the risks of inflammatory responses and CVD [10–12]. As the majority of older patients with diabetes consume low amount Ca and Mg, constructive strategies are needed to help these patients to achieve moderate Ca intake and adequate Mg intake through diet or supplements. Moreover, medical care practitioners should counsel patients on a more judicious dietary intake to avoid excess Ca consumption. In addition, the findings of the present study and those of previous studies suggest an optimal dietary Ca:Mg intake ratio of 2.0–2.5 for reducing CVD risks. RDA was established to meet the needs of 97%–98% of healthy individuals. According to RDA for the elderly population above 65 years of age, the Ca:Mg intake ratio ranges from 2.1 to 3.1 for men and from 2.4 to 4.1 for women [43–45]. Unfortunately, RDA provides an average value and does not establish the optimal balance of Ca:Mg intake ratio. Moreover, the majority of elderly people consume inappropriate amount of Ca and Mg, particularly, high amount of Ca and low amount of Mg through diet as well as Ca supplements, which could result in an inappropriate Ca:Mg intake ratio of >4, leading to an elevated risk of CVD. Hence, further studies on the current RDA for Ca and Mg are necessary.

### Limitations

While the results of the present study shed light on the effects of dietary Ca and Mg intakes on the risks of CVD in older patients with diabetes, there are several limitations to this study. First, the assessments of dietary intake and lifestyle data were highly dependent on the self-reported questionnaire. Therefore, overestimation, underestimation, or poor recall might have produced confounded results. Fortunately, these older patients with diabetes lived in rural areas, and thus, most of them had simple lifestyle and eating behaviors, which increased the effectiveness of the dietary survey. Second, the sample size was somewhat small, which may have reduced the statistical power of the subgroup analysis. Thus, to observe the effects of different dietary Mg and Ca intake levels on CVD risk, a larger sample size is required. Third, our current data showed that high or low Ca intake increased CVD risks, and suggested that moderate Ca and adequate Mg dietary intakes with a Ca:Mg intake ratio of 2.0–2.5 are important for preventing CVD in older patients with diabetes. These findings are in line with those reported by other studies [24,25]. The present findings may also be applicable to non-rural patient populations with type 2 diabetes and healthy adults. However, further studies are still needed to more accurately assess the role of dietary Mg and Ca intake in inflammatory response and CVD risk in other populations. In addition, further studies are required to establish whether a dietary intervention with optimal Ca and Mg intakes, as described earlier, would have a meaningful impact on CVD risks, and whether the effects of dietary Mg and Ca intakes on inflammatory stress and CVD risks could also be applied in the investigation of other diseases such as osteoporosis and cancer.

## Conclusions

High or low calcium intake was found to increase CVD risks in older patients with type 2 diabetes. A balanced dietary Mg and Ca intake is highly recommended, which could be a key in reducing inflammatory responses and CVD risks. Our data suggest that (1) consuming “moderate” amount of 402–600 mg Ca/day (approximately 67%–100% of Taiwan RDA for Ca), (2) consuming adequate amount of Mg (or meeting RDA for Mg for healthy individuals above 65 years of age), and (3) maintaining a Ca:Mg intake ratio of 2.0–2.5 are important for reducing CVD risks in older patients with diabetes.

## Consent

Written informed consent was obtained from the patient for the publication of this report.

## Abbreviations

ADA: American Diabetes Association
AI: Adequate intake value
BMI: Body mass index
Ca: Calcium
CVD: Cardiovascular disease
CRP: C-reactive protein
EAR: Estimated average requirement
eGFR: Estimated glomerular filtration rate
Mg: Magnesium
MDRD: Modification of diet in renal disease
NHANES: National Health and Nutrition Examination Survey
NAHSIT: Nutrition and Health Surveys in Taiwan
RDA: Recommended dietary allowance
RDW: Red blood cell distribution width
